# Preparation, Biological Evaluation and Dosimetry Studies of 175Yb-Bis-Phosphonates for Palliative Treatment of Bone Pain

**DOI:** 10.4274/mirt.36036

**Published:** 2015-11-02

**Authors:** Ashraf Fakhari, Amir R. Jalilian, Hassan Yousefnia, Saeed Shanehsazzadeh, Ali Bahrami Samani, Fariba Johari Daha, Mehdi Shafiee Ardestani, Ali Khalaj

**Affiliations:** 1 Tehran University of Medical Sciences, Faculty of Pharmacy, Tehran, Iran; 2 Nuclear Science and Technology Research Institute (NSTRI), Tehran, Iran

**Keywords:** Yb-175, pamidronic acid, alendronic acid, bone pain palliation therapy, biodistribution, dosimetry

## Abstract

**Objective:**

Optimized production and quality control of ytterbium-175 (Yb-175) labeled pamidronate and alendronate complexes as efficient agents for bone pain palliation has been presented.

**Methods:**

Yb-175 labeled pamidronate and alendronate (175Yb-PMD and 175Yb-ALN) complexes were prepared successfully at optimized conditions with acceptable radiochemical purity, stability and significant hydroxyapatite absorption. The biodistribution of complexes were evaluated up to 48 h, which demonstrated significant bone uptake ratios for 175Yb-PAM at all-time intervals. It was also detected that 175Yb-PAM mostly washed out and excreted through the kidneys.

**Results:**

The performance of 175Yb-PAM in an animal model was better or comparable to other 175Yb-bone seeking complexes previously reported.

**Conclusion:**

Based on calculations, the total body dose for 175Yb-ALN is 40% higher as compared to 175Yb-PAM (especially kidneys) indicating that 175Yb-PAM is probably a safer agent than 175Yb-ALN.

## Abstract (tr)

**Amaç:**

İterbiyum-175 (İb-175) işaretli pamidronat ve alendronat komplekslerinin kemik ağrısı palyasyonu için etkin ajanlar olarak optimal üretim ve kalite kontrolü sunulmaktadır.

**Yöntem:**

İb-175 işaretli pamidronat ve alendronat (175İb-PMD ve 175İb-ALN) kompleksleri kabul edilebilir radyokimyasal saflık, stabilite ve anlamlı hidroksiapatit emilimi ile optimize koşullarda başarıyla hazırlandı. Komplekslerin biyodağılımı 48 saate kadar değerlendirildi ve tüm zaman aralıklarında 175İb-PAM ile anlamlı kemik tutulum oranı saptandı. Aynı zamanda 175İb-ALN’nin çoğunlukla böbrekler yoluyla ekskrete edildiği tespit edildi.

**Bulgular:**

Bir hayvan modelinde, 175İb-PAM’ın performansı daha önce bildirilen diğer 175İb-kemiğe bağlanan kompleksler ile eşit ya da daha iyi olarak bulundu.

**Sonuç:**

Hesaplamalara göre, 175İb-ALN’nin için toplam vücut dozu (özellikle böbreklerde) 175İb-PAM’dan %40 daha yüksektir. Bu durum, 175İb-PAM’in 175İb-ALN’den daha güvenli bir alternatif olduğunu öne sürmektedir.

## INTRODUCTION

The incidence of bone metastasis is increasing mostly due to breast or prostate cancer, in other words primary bone cancer is becoming rare. Most of these patients suffer from severe pain that cannot be relieved by pharmaceuticals. Radiotherapy is an effective method for the treatment of tumor regions. However, in metastatic bone disease, the damaged regions are scattered, making radiotherapy an undesirable option. In most patients, chemotherapy is used in order to prevent malignant cell proliferation and cause cell death if the illness is in initial stages. In some cases, radiopharmaceuticals are valid choices for treatment (1). Beta emitters have already been used for the treatment and palliation of many types of cancer, particularly metastatic bone disease. For this purpose, an appropriate molecule that is absorbed into the bone as a ligand is labeled with a beta emitter radioisotope. The applied ligand can be a known drug or a new structure that can be absorbed into the bone. Many tetraphosphonates and bisphosphonates are being used for this purpose based on absorption of the phosphate group into the bone. When choosing radioisotopes, many features including the half-life, range and energy of beta particles should be taken into account. Numerous phosphonates were labeled with suitable radio lanthanide beta emitters and were used in clinical nuclear medicine for either therapy or palliation, due to the incidence of bone metastases in the world. For instance, preparation and application of 153Sm-EDTMP (2) as a tetraphosponate and 166Ho bisphosphonate is more outstanding (3). Bisphosphonates, a class of drugs that prevent loss of bony mass, are used to treat osteoporosis and similar diseases. Bone undergoes constant turnover and is kept in a balance (homeostasis) of osteoblasts creating bone and osteoclasts destroying bone. Bisphosphonates inhibit bone digestion by encouraging osteoclasts to undergo apoptosis, or cell death, thereby slowing bone loss. The two PO3 (phosphonate) groups that are covalently linked to carbon determine both the name “bisphosphonate” and the function of the drugs. Because of the importance of bisphosphonates among other bone pharmaceuticals, many agents within this class such as alendronate, pamidronate, etidronate, and zoledronic acid have been labelled with therapeutic radioisotopes such as 153Sm, 166Ho, 186Re or diagnostic radioisotopes and have been used in clinical practice so far.

175Yb can be produced by thermal neutron bombardment of natural ytterbium target. The simplified production scheme is: 174Yb (n, γ) 175Yb → 175Lu (Stable) σ=69 barn (Table 1) (4).

175Yb (T1/2=4.185 d) is an interesting radionuclide for targeted therapy modalities. Absorbed radiopharmaceutical remains in bone and acts as an internal generator. The beta energy is suitable that decay in bone without bone marrow separation (5).

The idea of developing bone avid agents based on ligands draws attraction due to the inhibitory binding affinity constant (Ki) of bisphosphonates used in clinical practice including pamidronate (83 μM) and alendronate (82 μM). In one study with 177Lu-zoledronate, the resulting complex was not stable in vivo as compared to other therapeutic bisphosphonates due to the existence of an imidazole ring (6). Thus, other ligands of interest based on their affinity constants and if they contain aliphatic amino chains were considered, such as pamidronic acid and alendronic acid.

**Figure 1:**
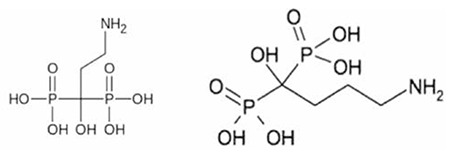
Chemical structure for pamidronic acid (left) and alendronic acid (right)

In this study, the 175Yb-alendronate and 175Yb-pamidronate complexes were prepared followed by preclinical evaluation including in vitro/vivo stability, and biodistribution studies by post-mortem studies. In continuation with previous experiences in the estimation of effective human dose extrapolation from rodents’ data (7,8,9), we attempted to compare the biodistribution data of intravenous (iv) injection of 175Yb-Alendronate (175Yb-ALN) and 175Yb-Pamidronate (175Yb-PAM), as well as a prediction of the human absorbed dose of each tracer that is derived from rat data (Figure 1).

## MATERIALS AND METHODS

The natural ytterbium oxide was purchased from Isotec Inc, USA and 175Yb was produced in the Tehran Research Reactor (TRR). Whatman No. 2 was obtained from Whatman (Maidstone, UK). Radio-chromatography was performed by using a Bioscan AR-2000 radio TLC scanner instrument (Bioscan, Paris, France). All other chemical reagents were purchased from Merck (Darmstadt, Germany). An approval was obtained from the Nuclear Science and Technology Research Institute Ethics Committee to conduct this research study. The Sprague Dawley rats were purchased from Pasteur Institute of Iran, Karaj, all weighed 20-25 g, were acclimatized at proper rodent diet and kept at 12h/12h day/night light/darkness.

### Production and Quality Control of 175YbCl3 Solution

Ytterbium-175 was produced by neutron irradiation of 1 mg of natural Yb2O3 at the neutron flux of 3×1013 n/cm2/s. Irradiation was carried out for 7d. The irradiated target was dissolved in 0.1 M HCl and the resultant solution was evaporated and was reconstituted in double distilled water. The radionuclidic purity of the solution was checked by using both high purity germanium (HPGe) spectroscopy for the detection of various interfering gamma emitting radionuclides and beta scintillation tests. Gamma spectroscopy of the final sample was carried out counting in an HPGe detector coupled to a Canberra TM multi-channel analyzer for 1000 s. The radiochemical purity of the 175YbCl3 was checked using two solvent systems by ITLC on Whatman No. 2 papers. A: 10% NH4OAc and methanol (1:1) and B: 10 mM DTPA solution (pH 5).

### Radiolabeling of Bisphosphonates with 175YbCl3

Stock solutions of mono sodium bisphosphonates salt were prepared by dissolution double distilled ultra pure water, to produce a solution of 50 mg mL-1 separately. For labeling, an appropriate amount of the 175YbCl3 solution containing the required activity (0.1 mL, 1850 MBq) was added to the desired amount of stock solutions (0.3 mL, 1:5; 1:10; 1:15; 1:20; 1:40 and 1:50 ratios for Yb: bisphosphonate). The pH of the mixtures was adjusted to 7. The complex solutions were kept at room temperature for 60-360 min. In addition, another set of experiment was performed at 60 °C warm bath for 60-360 min. The radiochemical purity was determined using ITLC. The final solution was passed through a 0.22-µm membrane filter and p H was adjusted to 7-8.5 with 0.05 molL-1 phosphate buffer (pH 5.5). A 5 mL sample of the final fraction was spotted on a chromatography Whatman No. 3 paper, and developed in Whatman 3 MM chromatography paper or ITLC-SG eluted with NH4OH (56%): MeOH (100%): H2O (100%) (0.2:2:4; v/v/v) as mobile phase mixture. Sterility was controlled with a random sampling following decay of radioactivity. The Limulus amoebocyte lysate (LAL) test was used for validation of radiopharmaceutical production according to the European protocol (10).

### Stability of 175Yb-Bisphosphonates in Final Formulation

Stability of 175Yb-bisphosphonates in the final preparation was determined by storing the final solution at 25 °C for up to 48 h and performing frequent ITLC analysis to determine radiochemical purity using Whatman 3 MM chromatography paper or ITLC-SG eluted with NH4OH (56%): MeOH (100%): H2O (100%) (0.2:2:4; v/v/v).

### In Vitro Protein Binding of 175Yb-Bisphosphonates in the Presence of Human Serum

In vitro protein binding of 175Yb-bisphosphonates was carried out in human blood by protein precipitation according to the published procedure (11). Three mL fresh human plasma was mixed with 1 mL of the labeled complex, and incubated for 1h at 37 °C. Contents of the tube were centrifuged at 3000 rpm for 10 min for separation of serum and blood cells. After adding approximately equal volume of 10% trichloroacetic acid (TCA), the mixture was centrifuged at 3000 rpm for another 10 min. The residue was separated from the supernatant and both layers were counted for radioactivity in a well type gamma counter. Protein binding of the complex was expressed as the fraction of radioactivity bound to protein, as percentage of the total radioactivity.

### In Vitro Stability of 175Yb-Bisphosphonates in the Presence of Human Serum

The final solution (200 µCi, 50 µL) was incubated in the presence of freshly prepared human serum (300 µL) (Purchased from Iranian Blood Transfusion Organization, Tehran) and kept at 37 °C for 2 days. Trichloroacetic acid (10%, 100 µl) was added every 30 min to a portion of the mixture (50 mL), and the mixture was centrifuged at 3000 rpm for 5 min followed by decanting the supernatant from the debris. The stability was determined by performing frequent ITLC analysis of the supernatant by using the aforementioned ITLC system.

### Hydroxyapatite Binding Assay

The hydroxyapatite binding assay was performed according to the previously described procedure (12), with only a slight modification. In summary, 2 mL of saline solution of pH 7.4 were added to vials containing 1.0, 2.0, 5.0, 10.0, 20.0 and 50.0 mg of solid hydroxyapatite, and the mixtures were shaken for 1 h. Then, 50 mL of the radioactive preparation was added and the mixtures were shaken for 24 h at room temperature. The suspensions were centrifuged, and two aliquots of the supernatant liquid were taken from each vial and radioactivity was measured with a well-type counter. Test experiments were performed using a similar procedure, this time in the absence of hydroxyapatite. The binding percentage of 175Yb to hydroxyapatite (HA) was calculated according to HB=1-A/Bx100, where A is the mean radioactivity value of the supernatant sample being studied and B is the mean total value of whole activity used.

### Biodistribution in Sprague Dawley® Rats

During the entire study, autoclaved food and drinking water were available ad libitum. Animal studies were carried out in accordance with the UK Biological Council’s Guidelines on the Use of Living Animals in Scientific Investigations, 2nd edition (approved by Iranian Ministry of Health and Medical Education) (11). To determine its biodistribution, the radiotracer was administered to Sprague Dawley® rats that were purchased from Razi Institute, Karaj, Iran. One hundred µL of final radiotracer solution containing 7-7.4 MBq radioactivity was injected intravenously to rats through their tail vein. The total amount of radioactivity injected into each animal was measured by counting the 1-ml syringe before and after injection in a dose calibrator with fixed geometry. The animals were sacrificed using the animal care protocols at selected times after injection (2 to 48 h), the tissues (blood, heart, lung, small intestine, large intestine, skin, stomach, kidneys, liver, muscle and bone) were removed, washed with normal saline and dried in paper toweling (12). Each sample was weighed and counted with an HPGe detector 3 times, along with standards prepared from a sample of the injected material. The percentage of injected dose per gram (ID/g %) of each organ was measured by direct counting from 11 harvested rat organs (based on the area under 396.33 keV peak obtained by an HPGe detector).

### Measurement of Activity

All samples were background subtracted, the decay correction was not performed, and similar samples were averaged together. For each of these measurements, three samples (from each organ) were weighed and then counted by HPGe to determine the percentage of injected dose per gram (which was equivalent to the percentage of injected activity per gram %IA/g≡%ID/g); all the organ activity measurements were normalized to injected activity (13). In all measurements, we tried to keep the same geometry and same volume in order to prevent overestimation and underestimation in dose measurements (14). Uncertainties in the determinations were minimal, because each assay collected at least 10,000 counts, which resulted in a standard deviation (SD) of less than 1%. All samples were background subtracted and decay correction was not considered for all measurements, and the similar samples were averaged together. The activity in the syringes was measured before and after administration of the radiopharmaceutical with well-type ionization chamber (CRC-15R, Capintec, USA N.J.).

The HPGe detector provides us a count, therefore we used the below formula to convert these counts into activity measure (15):

(Eq.1)

Where t is the time of counts and Eff is the efficiency of the detector for the selected energy and Br is the decay yield of selected energy (396.33 keV) for 175Ytterbium.

The 175Yb activity concentration at time t, %ID/g (t), was then calculated as the percentage of injected activity per gram of tissue (%IA/g):

(Eq.2)

Where Atissue is the 175Yb activity in the sample, Mtissue is the mass of the sample and Atotal is the total activity of 175Yb injected into the rat (15,16).

The cumulated activity in the source organs, can be calculated by equation 3:

(Eq.3)

Where Ah(t) is the activity of each organ at time t (16). In this study, the accumulated source activity was calculated by plotting the non-decay corrected time activity curves for each organ and computing the area under the curves. For this purpose, the data points which represent the percentage-injected dose were created and fitted to a mono-exponential, bi-exponential or uptake and clearance curve (17). In addition, the curves were extrapolated to infinity (5 times higher than physical half-life of 175Yb) by fitting the tail of each curve to a mono-exponential curve. Then the area under the curve was calculated in the same way as explained in a previous study by our group on determination of the absorbed dose of 67Ga-DTPA-ACTH (18). In order to prevent under-sampling for the area under the curves (AUCs), we tried to calculate the AUCs when the R2 square of fitted curves were above 0.9 for each organ. We used the mentioned criteria, and included 11 organs for AUCs calculation as source organs for MIRD.

### Extrapolation of the Organ Uptake Data to Humans

The organ uptake data in animals were used to extrapolate the equivalent uptake in humans by the proposed method of Sparks and Aydogan (19) as represented in equation 4.

(Eq.4)

### Absorbed Dose Calculations

The absorbed radiation dose was calculated by MIRD formulation (19):

(Eq.5)

Where D (rk) is the absorbed dose of the target organ, is the accumulated activity in source organs, and called the S factor is defined as the mean absorbed dose to the target region rk per unit accumulated activity in the source region rh. The S factor represents the physical decay characteristics of radioisotopes, the range of emitted radiations, and the organ size and configuration expressed in (mSv/(MBq*s)) (20). The S factors have been taken from the tables presented in the Medical Internal Radiation Dose No.11 also available in http://doseinfo-radar.com/RADARphan.html (20).

### Calculation of Effective Absorbed Dose

The effective absorbed dose of each organ is calculated by the expression:

(Eq.6)

Where HT is the equivalent dose in a tissue or organ, while T and WT represent the tissue-weighting factor according to ICRP 106 (21).

## RESULTS

### Production of 175Yb

Around 1.5-2 GBq/g of 175Yb activity was obtained after 7 days of irradiation at a flux of 3×1013 n/cm2/s using natural Yb2O3 target. A thorough study on the various irradiation times and neutron fluxes was performed for radionuclide production to achieve an optimized condition determination. Major radionuclidic impurities in irradiated samples were shown to be. 169Yb and 177Lu based on time of irradiation.

**Figure 2:**
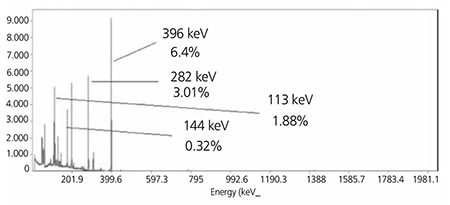
Gamma spectrum for 175YbCl3 solution used in radiolabeling

The observed gamma-photo peaks correspond to the gamma-photo peaks of 175Yb (113, 144, 286 and 396 keV), 169Yb (63, 110, 130, 177, 198, 261 and 307 KeV) and 177Lu (208 and 250 keV). By analyzing the gamma-ray spectra, the radionuclidic purity of 175Yb was found to be 96.2% with the presence of 2.1% 169Yb. Beta spectrum of the final sample used in radiolabeling was also obtained showing a maximum peak around channel 450. The calculated specific activity of Yb-175 was 600-800 GBq/mmol (Figure 2).

**Figure 3:**
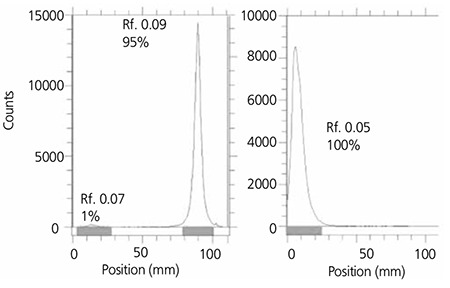
ITLC chromatograms of 175YbCl3 solution in 10 mM DTPA solution (pH~4) (left) and in 10% ammonium acetate: methanol (1:1) (right) on Whatman No. 1 Paper

For radiochemical purity, two solvent systems were used. In a mixture of 10% NH4OAc:methanol (1:1), the free cation remains at the base while any undistinguished anions would migrate to higher Rfs (not observed). On the other hand in 10 mM DTPAsolutionYb-175 cation is complexed in 175Yb-DTPA form migrating to higher Rfs and any possible colloidal fraction would remain at the base (Figure 3).

**Figure 4:**
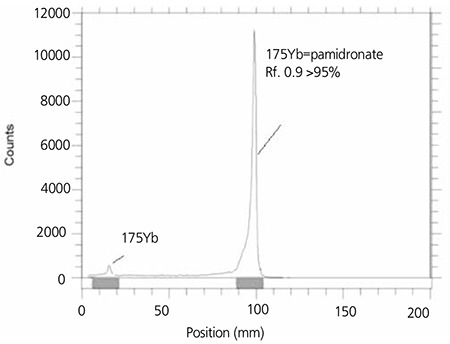
ITLC chromatogram 166Ho-Pamidronate solution using NH4OH: MeOH: H2O (0.2:2:4)

The radiochemical purity in 10 mmol L-1 DTPA aq. solution (solvent 1), free 175Yb3+ cation is complexed to more lipophilic 175Yb-DTPA form and migrates to higher Rf. The small radioactive fraction remaining at the origin could be related to other Yb ionic species that do not form Yb-DTPA complex, such as YbCl4- and/or colloids. On the other hand, 10% ammonium acetate:methanol mixture (1:1) (solvent 2) was used for determination of radiochemical purity. The fast eluting species was Yb3+. Other ionic forms of 175Yb such as YbCl4- as well as colloids remained at the origin (Rf=0) (Figure 4).

### Labeling Optimization Studies:

In order to obtain maximum complex yields, several experiments were carried out with different reaction parameters such as ligand concentration, pH, reaction time and temperature. Ligand concentration varied between a wide range starting from 10 to 50 mg/mL for bisphosphonates. At optimum optimized conditions, 175Yb-ALN was obtained with a specific activity of 3.7±0.2 GBq/mmol while it was 4.1±0.2 GBq/mmol for 175Yb-PAM.

**Figure 5:**
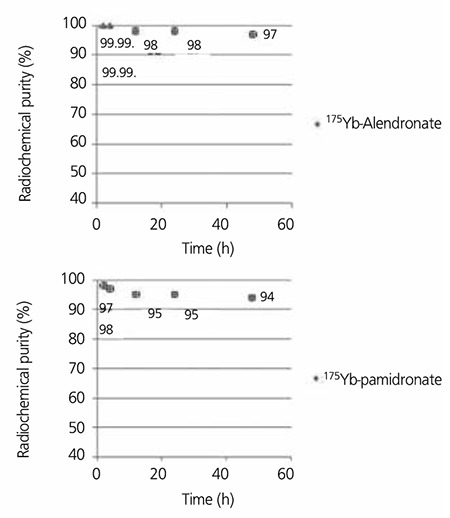
Stability studies for 175Yb-Alendronate (up) and 175Yb-Pamidronate in the final formulation up to 48 h

It was observed that at room temperature >95% complex was achieved with 15 mg/mL of bisphosphonates. The best ITLC mobile phase was evaluated by Whatman No.2 paper using NH4OH: MeOH: H2O (0.2:2:4) as shown in Figure 5.

The effect of various factors such as ligand concentration and temperature on labeling yield of 166Yb-PMD were also studied. The results are shown in Table 2. Labeling yield increased with increasing molar ratio Yb:PMD (from 1:5 to 1:50) and reached to more than 99% in 60 minutes (Table 1). The stability of prepared 175Yb-complexes was checked up to 48 hours after preparation. The complex was stable in final pharmaceutical sample and its radiochemical purity was above 99% even 48 hours after preparation using Whatman 3 MM eluted with NH4OH: MeOH: H2O (0.2:2:4).

### Stability Studies and Protein Binding

Stability test was developed for the complex in the presence of human serum at 37 °C using ITLC as mentioned above, and all data within 48 h were above 89% at all-time intervals (22). It was identified that protein binding using ITLC of the serum-radiopharmaceutical mixture was 57%, while 43% was found in the free form in the circulation. The protein binding for the PMD ligand has been reported in different references as 54% in the free form (12), however, there was no data on protein binding for metal PMD complexes in the literature.

### Hydroxy Apatite Binding Assay

**Figure 6:**
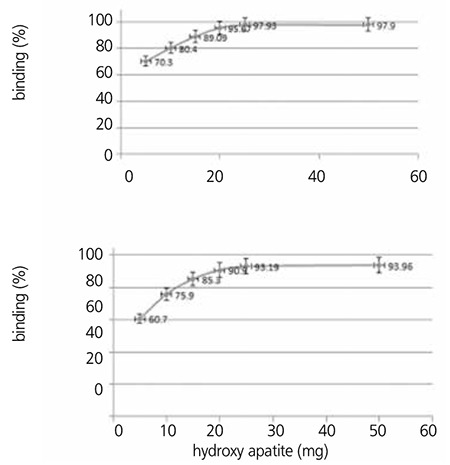
Hydroxy apatite binding assay data for 175Yb-Alendronate (up) and 175Yb-Pamidronate (down) at 37 °C in 24 h

**Figure 7:**
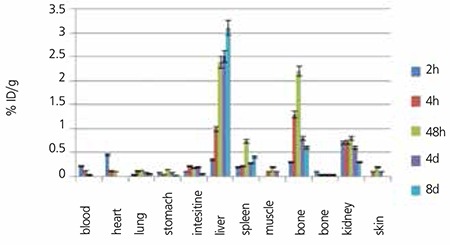
Biodistribution of 175YbCl3 (1.85 MBq, 50 mCi) in wild-type mice 2, 4, 48 h and 4, 8 days after iv injection via tail vein (ID/g%: percentage of injected dose per gram of tissue (n=5)

HA assay demonstrated high capacity binding for 175Yb-ALN to hydroxy apatite. More than 90% binding was observed even at 4 mg of HA, while at 6 mg HA binding was obtained in >95% (Figure 6). On the other hand, for 175Yb-PAM more than 90% binding was also observed at 4 mg of HA, while at 6 mg HA there was >99% binding (Figure 7).

### Biodistribution

**Figure 8:**
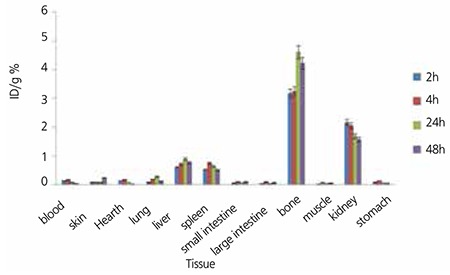
Percentage of injected dose per gram of 175Yb-Pamidronate in wild-type rat tissues at 2, 4, 24 and 48 h post injection (n=3)

For better comparison of biodistribution, the study was performed for free Yb3+ as well. The %ID/g data are summarized in Figure 8. For free Yb3+ cation, the radioactivity was mainly located in the liver, kidney and bone. The free cation is soluble in water and it can be excreted via the urinary tract.

Since the metallic Yb3+ is transferred in the plasma as protein-bond form, the major final accumulation was shown to be in the liver reaching >3% after 8 days. Based on bio-equality of lanthanide cations with calcium ions, the Yb3+ is also absorbed on hydroxyl apatite texture of the bone; thus, a 2.25% bone uptake is observed in 48 hours.

The liver radioactivity uptake of the cation is comparable to other radio-lanthanides such as Yb, Sm and Tb (23). About 2.5% of the cation radioactivity accumulates in the liver in 48 h. Low blood radioactivity content demonstrates the rapid removal of 175Yb from the circulation after injection. The lung, muscle and skin do not demonstrate significant 175Yb uptake, in accordance with other cation accumulation patterns. A low bone uptake is observed for 175Yb that remains almost constant after 48 h (0.7%). Bone uptake of Yb-175 is greater than those of the agents under examination, which is also the case for other radiolanthanides since they accumulate based on Ca-cation resemblance. However, the major issue in all free lanthanides is the high liver uptake as observed for Sm-153 cation or Sm-153 EDTMP. The main issue in bone avid agents is their Bone: liver uptake ratio.

Spleen also has significant 175Yb uptake possibly related to the reticuloendothelial system. The free cation is soluble in water and it can be excreted via the urinary tract.

The radioactivity biodistribution of 175Yb-PMD (200 µCi in 150 µL volume) in rat organs upto 48 h post-injection was determined, and it was clearly shown that the major portion of the injected 175Yb-PMD radioactivity was transferred from the blood circulation into the bones (Figure 8). The significant radioactivity excretion observed in kidneys was anticipated due to the major PMD non-metabolized excretion through the kidneys, that has been already reported (19). As compared to other 175Yb bone seeking agents, i.e. 175Yb-DOTMP with less than 3% bone uptake in 48 h (24), 175Yb-PAM demonstrated better uptake within the same period. On the other hand, the bone uptake reported for another Yb-175 complex, 175Yb-EDTMP (4.7 at 24 h) is very similar to that of 175Yb-PAM (4.6 at 24 h) (25).

The report denoted that after administration of PMD, 46±16% (overall mean ± SD) of the parent drug was excreted unchanged in the urine within 120 hours. The cumulative urinary excretion showed linear correlation with dose. The mean ± SD elimination half-life was 28±7 hours. The mean ± SD total and renal clearances of pamidronate were 107±50 mL/min and 49±28 mL/min, respectively. The rate of elimination from bone has not been determined.

It can be suggested that a mild diuretic agent would remove the un-concentrated portion of the radiopharmaceutical into bones from the circulation through urinary tracts, and possibly enhance the bone: non-target ratio for the therapeutic radiopharmaceutical leading to unwanted irradiation doses to the patients (esp. gonads). The liver does not play a significant role in metabolism (<1%), and also lower GI (intestines, colon) uptake is observed.

**Figure 9:**
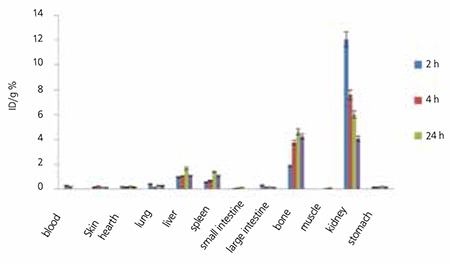
Percentage of injected dose per gram of 175Yb-Alendronate in wild-type rat tissues at 2, 4, 24 and 48 h post injection (n=3)

175Yb-ALN also demonstrates significant bone uptake at all-time intervals, especially 24 h after injection while high kidney uptake is observed at all-time intervals that could be due to urinary excretion of the complex. Urinary excretion rate of 175Yb-ALN was higher as compared to 175Yb-PAM that has a slightly higher liver uptake. Similarly, the use of a diuretic could reduce the absorbed dose form the kidney to vital organs (Figure 9).

The target: non-target ratio of 175Yb-PMD was determined in order to demonstrate the targeting property of radiopharmaceutical 2-48 h post injection as shown in Table 3. As shown in the table, best targeting can be observed 48 h post injection (almost half a physical half-life) in the blood, kidney and liver. Interestingly, the same trend has been observed for Ho-166 analogue of this phosphonate (26).

**Figure 10:**
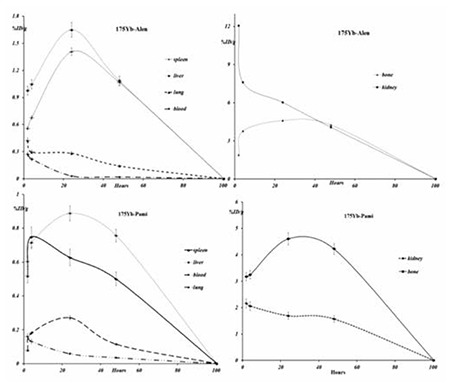
The non-decay corrected clearance curves from each rat organ. The x-axis is displayed as hours. Data are presented as mean ± SD and the mean values as the percentage of administered activity per gram (%ID/g)

Also in case of 175Yb-ALN as shown in the Table 4, the best targeting can be observed in the blood, kidney and liver 48 h post injection (almost half a physical half-life). However, the biological affinity of this complex into bones is significantly less than the pamidronate analog. Figure 10 demonstrates the non-decay corrected clearance curves from each rat organ after i.v. injection of 175Yb-PAM and 175Yb-ALN.

### Dosimetry

As represented in Table 4, i.v. injection of 175Yb-PAM into humans would result in an estimated absorbed dose of 19.9 µSv/MBq according to prediction based on rat data. While the highest effective absorbed dose for 175Yb-PAM were in the osteogenic cells and kidneys (26.9 µSv/MBq), other organs receiving high doses were the spleen (20.2 µSv/MBq), red marrow (7.9 µSv/MBq) and the heart wall (6.8 µSv/MBq) in decreasing order. On the other hand, due to the deference in cumulative activity in the kidneys, our estimation shows that i.v. injection of 175Yb-ALN into the humans would result in an estimated absorbed dose of 28 µSv/MBq. The highest effective absorbed dose for 175Yb-PAM were detected in the kidneys (84.5 µSv/MBq) followed by the spleen (38.7 µSv/MBq), osteogenic cells (27.6 µSv/MBq) and the heart wall (8.5 µSv/MBq).

Discovering a new radiopharmaceutical agent requires assessment of its biodistribution in nonhuman models prior to its clinical application, most commonly rats, based on the assumption that biodistribution will be similar in rats and in man (27), which is a common first step consistent with the recommendations of ICRP 103 (28).

Although we did not measure the rat’s whole body activity at each time point, the prediction can be accepted as precise and close to practical absorbed dose values since activities of more than 11 organs were used in this study. There is another limitation for using the methods described above: the issue in selecting the time points. It would have been better to have more time points before 2 hour to avoid underestimation. Therefore, further studies are required to assess the exact value of cumulative activity in the bladder; however, due to the same kinetics of both radiotracers, it is not expected that results would significantly differ.

The prediction of human dose based on rodent’s data is not as simple as we explained, and using extrapolation from animal data may lead to some over or under estimations. Allowable doses based on human data were somewhat different from those obtained from studies of rodents as well as nonhuman primates Table 5 and Figure 10 compare the increments in human absorbed dose after injection of 175Yb-PAM versus 175Yb-ALN (29,30). As indicated in Table 4, the total body dose for 175Yb-ALN was 40% higher as compared to 175Yb-PAM. Moreover the received kidney dose was 3 times higher; implicating that 175Yb-PAM is more suitable and safer than 175Yb-ALN.

## CONCLUSION

175Yb-PMD and 175Yb-ALN were prepared (radiochemical purity>95%) using optimization studies. 175Yb-PMD, 175Yb-ALN and 166HoCl3 preparations were administered intravenously through the tail vein to rats and biodistribution data was checked 2 h to 48 h later, showing at least 72% accumulation of 175Yb-PMD in bony tissues. Satisfactory stability was obtained in the presence of human serum and final formulations. HA binding assay demonstrated >95% binding with 4-6 mg of HA in 24 h at 37 °C. The complex protein binding was about 50-55%. The high bone uptake ratios at all time intervals for 175Yb-PMD was in accordance with the HA test. The bone uptake ratios at all time intervals were obtained. The bone: kidney, bone: blood and bone: liver uptake ratios were significantly higher for 175Yb-PMD at 48 h post injection (2.69, 5.58 and 120 respectively). 175Yb-PAM demonstrated better uptake at the same time interval as compared to 175Yb-DOTMP (<3% bone uptake in 48 h). On the other hand, the bone uptake reported for 175Yb-EDTMP (4.7 at 24 h) is very similar to that of 175Yb-PAM (4.6 at 24 h). Based on calculations, the total body dose for 175Yb-ALN is 40% higher as compared to 175Yb-PAM (especially kidneys) indicating that 175Yb-PAM is a possible safer agent than 175Yb-ALN.

## Figures and Tables

**Table 1:**
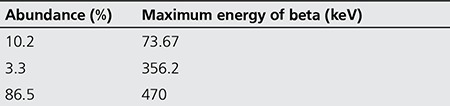
Beta decay characteristics for Yb-175 radionuclide

**Table 2:**
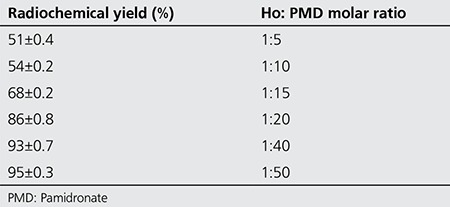
The effect of various molar ratios of Ho and pamidronate on the radiochemical yield (30 min at pH. 8-9) (n=3)

**Table 3:**
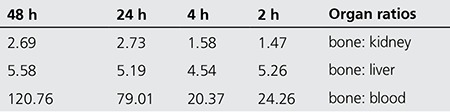
Target (bone)/non target ratios for 175Yb-Pamidronate at various time intervals in rats organs

**Table 4:**
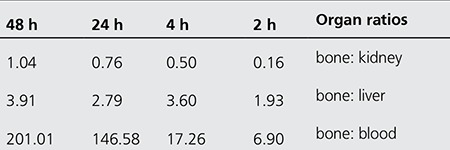
Target (bone)/non target ratios for 175Yb-Alendronate at various time intervals in rats organs

**Table 5:**
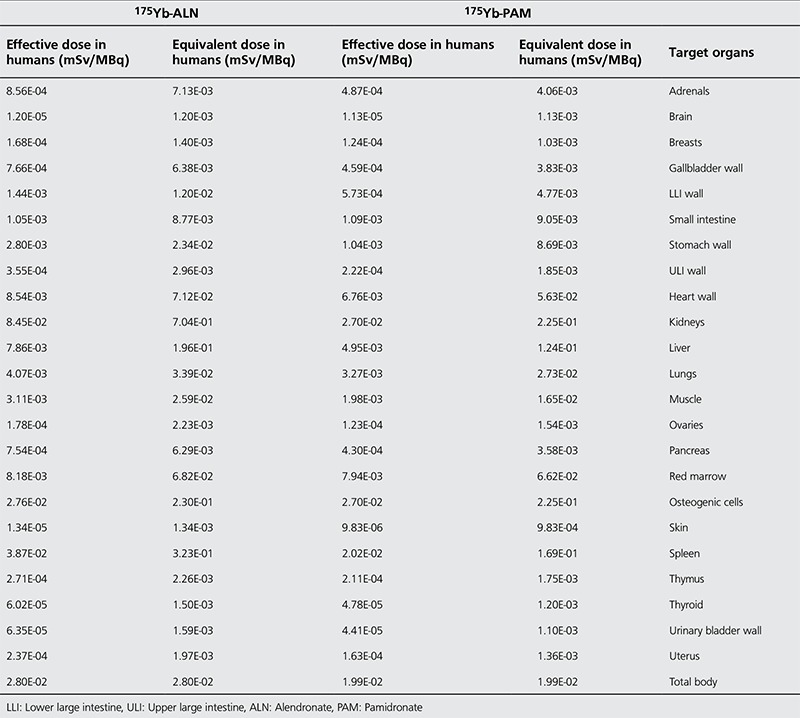
Prediction of human absorbed dose based on rat data after i.v. administration of 175Yb-Pamidronate and 175Yb- Alendronate

**Figure 11:**
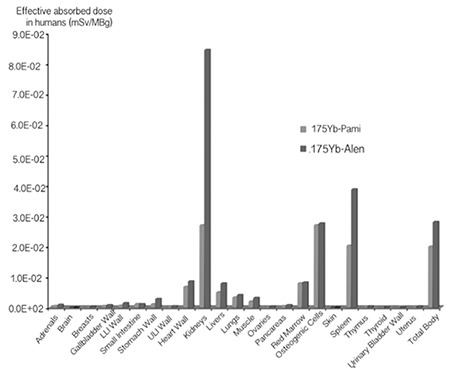
Comparison of effective absorbed dose prediction in humans based on rat data after i.v. injection of 175Yb-Pamidronate and 175Yb-Alendronate
